# The role of secreted proteins in efferocytosis

**DOI:** 10.3389/fcell.2023.1332482

**Published:** 2024-01-08

**Authors:** Guangsheng Hou, Xinyu Wang, Anhua Wang, Lei Yuan, Qian Zheng, Hui Xiao, Hui Wang

**Affiliations:** College of Life Sciences, Shaanxi Normal University, Xi’an, China

**Keywords:** apoptosis, apoptotic cell clearance, efferocytosis, secreted proteins, bridging molecule

## Abstract

The clearance of apoptotic cells known as efferocytosis is the final stage of apoptosis, and includes the recognition, phagocytosis, and degradation of apoptotic cells. The maintenance of tissue homeostasis requires the daily elimination of billions of apoptotic cells from the human body via the process of efferocytosis. Accordingly, aberrations in efferocytosis underlie a growing list of diseases, including atherosclerosis, cancer, and infections. During the initial phase of apoptosis, “Eat-Me” signals are exposed and recognized by phagocytes either directly through phagocyte receptors or indirectly through secreted proteins that function as bridge molecules that cross-link dying cells to phagocytes. Here, we set out to provide a comprehensive review of the molecular mechanisms and biological significance of secreted proteins in apoptotic cell clearance. Specifically, it focuses on how these secreted proteins act as bridging molecules to facilitate the clearance process.

## 1 Introduction

Cell death is as crucial as cell division in maintaining an organism’s normal growth, development, and life activities, and billions of cells in the human body die every day through apoptosis. The clearance of apoptotic cells is the final stage of apoptosis, which includes the recognition, phagocytosis, and degradation of apoptotic cells (Adachi-Yamada et al., 1999; Morioka et al., 2018). Defective clearance of apoptotic cells triggers a variety of chronic inflammatory diseases such as systemic lupus erythematosus, rheumatoid arthritis, glomerulonephritis, and atherosclerosis (Elliott and Ravichandran, 2010; Ramirez-Ortiz et al., 2013; Han et al., 2016; Henson, 2017). There are numerous proteins implicated in the elimination of apoptotic cells. Among these, the secreted proteins have been attracting increasing interest (Savill and Fadok, 2000). Proteins secreted from apoptotic cells or macrophages play an important role in efferocytosis (Krispin et al., 2006; Zhang et al., 2012; Nichols et al., 2016). In this review, the mechanisms and research progress of secreted proteins in efferocytosis were discussed.

## 2 Apoptotic cell clearance

Apoptosis can be divided into several separate stages, including death signal initiation and transmission, death program activation, and the removal of apoptotic cells (deCathelineau and Henson, 2003). The extrinsic death receptor pathway and intrinsic mitochondrial pathway are the two major apoptotic pathways that activate the caspase protease family, whereas the inhibitor of apoptosis (IAP) family (XIAP, cIAP1, C-IAP2, NAIP, Livin and Survivin) prevents apoptosis by inhibiting the activity of various caspases (Silke and Meier, 2013; Silke and Vucic, 2014). The extrinsic route is induced by the interaction between death receptors and ligands. The TNFR family (TNFR1/2, Fas, and DR3/4/5) and related ligands (TNF FasL, TRAIL, and TWEAK) are death receptors that, upon activation, stimulate the assembly of the death-inducing signaling complex (DISC) and activate caspase-8 and caspase-3 (So and Ishii, 2019). The intrinsic pathway is a result of environmental duress, dysfunctional mitochondria, and DNA damage. Mitochondrial damage modifies the permeability of the mitochondrial outer membrane, resulting in the release of cytochrome c and its association with apoptotic protease activating factor-1 (Apaf-1), which triggers the caspase cascade reaction (Dehkordi et al., 2022).

When cells die via apoptosis, the removal of apoptotic cells can be divided into several principal steps. The first step requires the release of find-me signals by apoptotic cells, which assist in attracting phagocytes to the regions of tissue death (Tajbakhsh et al., 2022). The second phase is the exposure of eat-me signals on the surface of apoptotic cells, which facilitates specific recognition by phagocytes and subsequent uptake of the corpse (Furuta and Zhou, 2023). The third phase is the processing of the ingested cargo by the phagocyte, during which the ingested corpse undergoes a series of phagosome maturation steps that ultimately result in its decomposition (Underhill, 2005).

### 2.1 “Find-me” signals in apoptotic cell clearance

Dying cells release redundant “find me” signals, such as phospholipids (LPC, S1P), nucleotides (ATP, UTP), and chemokines (CXCCL1), to recruit phagocytes (Isfort et al., 2011). The first “Find-Me” signal is LPC. Its release is dependent on caspase-3 activating calcium-independent phospholipase A2, which degrades membrane phosphatidylcholine. On phagocytes, the nucleotides stimulate the purine-dependent receptor P2Y(2). S1P promotes apoptotic cell clearance and immune tolerance by activating erythropoietin signaling in macrophages. Some secreted proteins, such as cytokines and chemokines, function as “Find-Me” signals during the phagocytosis of apoptotic cells during apoptotic cell clearance (Kourtzelis et al., 2020; Tajbakhsh et al., 2022). In addition to eliciting apoptosis, Fas/CD95 stimulation has been shown to activate a pro-inflammatory program that results in the expression of numerous cytokines and chemokines. Expression and secretion of a number of cytokines and chemokines, such as MCP-1 and IL-8, prompt phagocytosis of apoptotic cells (Singh et al., 2021; Schimek et al., 2022).

### 2.2 “Eat-me” signaling during apoptotic cell clearance

When macrophages approach or come into proximity to apoptotic cells, exposure of the “Eat-Me” signal transform into a phagocytic signal that is recognized by phagocytic receptors or specific bridging molecules on the phagocyte, thereby initiating a phagocytic response. The “Eat-Me” signal has been investigated with phosphatidylserine (PS) the most. Phospholipids are distributed asymmetrically between the inner and outside leaflets of the plasma membrane of eukaryotic cells, with phosphatidylserine (PS) located in the cytoplasmic membrane’s inner leaflet. The asymmetric distribution of phospholipids is regulated by phospholipid translocases (Fippases) and phospholipid flipping enzymes (Scramblases) (Tanaka et al., 2011; Jensen et al., 2017). By propelling its own activity through ATP, phospholipid translocases contributes significantly to the maintenance of the asymmetric distribution of phospholipids in biological membranes by limiting phosphatidylserine molecules to the intracellular side of the membrane (Nagata et al., 2016). Phospholipid flippase enzymes rapidly exposes phosphatidylserine to the surface of apoptotic cells by rapidly disrupting the phospholipids between lipid bilayers and thereby exposing phosphatidylserine (Nagata et al., 2020). In apoptotic cells, the inner PS is exposed to the outer side of the phospholipid bilayer. This inside-out flipping process is dependent on the PS scramblase (Xkr8) (Nagata and Segawa, 2021). It is commonly recognized that exposure to phosphatidylserine (PS) in the exoplasmic leaflet transduces vital signals for platelet coagulation and apoptotic cell clearance (Bai et al., 2020). The reverse translocation of PS from the inner to the outer leaflet of the plasma membrane re-quires the action of flippases, and current research suggests that the major flippases are the calcium-dependent transmembrane protein TMEM16 and the XKR family, both of which are activated by caspase excision (Pedemonte and Galietta, 2014; Suzuki et al., 2014). Among them, the XKR family protein Xkr8 mediates PS externalization and promotes phagocytosis of dead cells in an evolutionarily conserved manner (Suzuki et al., 2014). Other signaling changes, such as calreticulin release from the endoplasmic reticulum of dying cells, externally exposed chromatin and DNA, and changes in cell surface charge and glycosylation, also serve as “Eat-Me” signals for the phagocytic recognition process. There are “don’t-eat-me” signals in addition to “Eat-Me” signals. CD47 inhibits “Eat-Me” signals like IgG and phosphatidylserine, which inhibit phagocytosis and protect healthy cells from phagocytosis (Russ et al., 2018). The IgG and phosphatidylserine “Eat-Me” signal inhibits phagocytosis and protects healthy cells against phagocytosis. In addition, tumor-expressed CD24 promotes immune evasion by interacting with the inhibitory receptor sialic acid-binding Ig-like lectin 10 (Siglec-10) expressed as an anti-phagocytic signal by tumor-associated macrophages (TAMs) (Barkal et al., 2019; Cheng et al., 2021).

### 2.3 The ingested cargo by the phagocytes in apoptotic cell clearance

Model organisms (*C. elegans* and *Drosophila*) as well as mammals have been used to study how phagocytes integrate signals from apoptotic cells to drive cytoskeletal rearrangement. Upstream signals in *C. elegans* were discovered to converge in two parallel and independent signaling pathways:the CED-2, CED-5, and CED-12 pathway, and the CED-1, CED-6, and CED-7 pathway (Kinchen et al., 2005). Both pathways then activate CED-10, an evolutionarily highly conserved GTPase, which in turn stimulates skeletal rearrangement to form phagocytic vesicles. When it comes to regulating the clearance of apoptotic cells, the CED-2/CED-5/CED-12 homologous signaling routes in *Drosophila* and mice were CG1587/myoblast city/Dmel, RKII/Dock180/ELMO1(Kinchen et al., 2005), and the CED-1/CED-6 homologous signaling pathways were Drpr/dCed-6 (Nakano et al., 2019), MEGF10/GULP1 (Morizawa et al., 2017). Under the influence of two pathways, phagocytes extend pseudopods for the envelopment of apoptotic cells to form phagosomes. Following maturation by a succession of Rab GTPases, phagosomes fuse with lysosomes to form phagolysosomes. Finally, apoptotic cell fragments are degraded by lysosomal membrane proteins LAAT-1 and a variety of lysosomal hydrolases, such as cathepsin L1(CPL-1) and type II DNA lyase (NUC-1) (Morizawa et al., 2017). This also reveals that some of the molecules affecting apoptotic cell clearance are evolutionarily highly conserved.

## 3 Secreted proteins in apoptotic cell clearance

Secreted proteins mostly act as bridging molecules that facilitate phagocytic identification during the removal of apoptotic cells. The bridge molecule binds to the “Eat-Me” signal and improves apoptotic cell detection and phagocytosis by connecting apoptotic cells with phagocytic receptors. Not all secreted proteins increase phagocytosis. After establishing a communication link between the “Eat-Me” signal and the phagocytic receptor, some released proteins inhibit phagocytosis of apoptotic cells. Furthermore, activation of receptor-mediated signaling pathways in macrophages increases the release of a number of anti-inflammatory substances, which not only aid in the resolution of inflammation but also improve macrophage phagocytosis after the release specific cytokines acting on them.

### 3.1 *C. elegans* secreted proteins in apoptotic cell clearance

**FIGURE 1:**
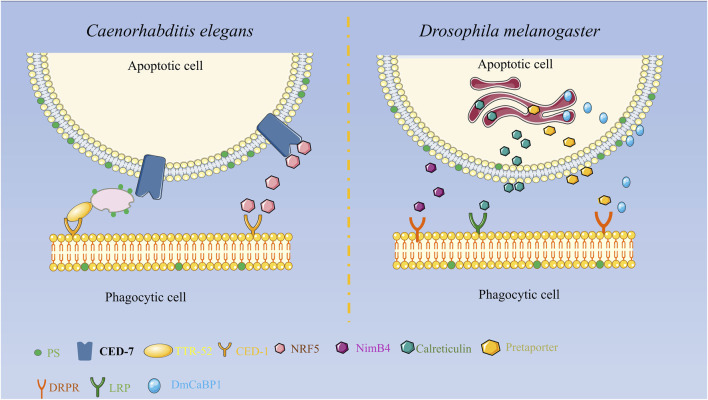
Diagrammatic representation of the secreted proteins’ roles in apoptosis clearance in *Drosophila melanogaster* and *C. elegans*. In *Drosophila melanogaster*, Pretaporter interacts to the phagocyte receptor Draper as a ligand, NimB4 recognizes receptors in a PS-dependent manner, calreticulin is recognized by macrophages as a phagocytic signal, and DmCaBP1 serves as a bridge between apoptotic cells and phagocytes. Functions as a linker to join phagocytes and apoptotic cells.

Some reports have discovered that TTR-52 is expressed and secreted in nematode endoderm and cells surrounding apoptotic cells and is involved in the CED-1, CED-6, and CED-7-mediated cell corpse phagocytosis pathway. TTR-52 influences the phagocytic receptor CED-1 to accumulate around apoptotic cells, which can act in parallel with other known phosphatidylserine receptors. CED-7 and TTR-52 may promote PS efflux from extracellular cells via extracellular PS vesicle production and thus PS expression on phagocytic cells via TTR-52 and CED-1, thereby facilitating the clearance of apoptotic cells. It was discovered that the secretory protein NRF-5 may cooperate with CED-7 and TTR-52 to promote phagocytosis by mediating the transmission of PS from apoptotic cells to phagocytes as shown in Figure 1 (Tan et al., 2010; Zhang et al., 2012; Nichols et al., 2016).

### 3.2 *Drosophila melanogaster* secreted proteins in apoptotic cell clearance

Externalization or surface exposure of endoplasmic reticulum proteins is a characteristic of apoptotic cells. The research found several endoplasmic reticulum proteins in *Drosophila melanogaster* that function in apoptotic cell clearance. Calreticulin, a soluble protein of approximately 46 kD in size, is extensively distributed in cells and plays a significant role in biology. Calreticulin participates in calcium homeostasis and functions as a protein chaperone in the endoplasmic reticulum. It functions as a recognition ligand at the cell surface by binding and activating low-density lipoprotein receptor-related protein (LRP) on phagocytes. Calreticulin’s movement to the cell surface may be associated with other events occurring on the surface of apoptotic cells, including membrane blistering, PS externalization, and structural alterations in lipid rafts, among others. When apoptosis occurs, calreticulin forms aggregates on the cell surface, which facilitate the recognition and phagocytosis of apoptotic S2 cells by *Drosophila* hematogenous l (2)MBN cells. Calreticulin is the first phagocytic marker to be identified in *Drosophila* (Gardai et al., 2005; Kuraishi et al., 2007; Krysko et al., 2018). Pretaporter (PRTP), a *Drosophila* endoplasmic reticulum protein with 416 amino acid residues, has three structural domains that resemble thioredoxins: an ER-retaining motif at the C terminus and an N-terminal signal peptide. PRTP is often found in the endoplasmic reticulum lumen and is frequently expressed throughout development. As a ligand, PRTP interacts with the phagocytic receptor Draper during apoptosis and stimulates tyrosine phosphorylation, which in turn activates the Rac1/Rac2 signaling pathway and causes phagocytic cytoskeletal reorganization and phagocytosis (Kuraishi et al., 2009). While researching the phagocytic receptor Draper in *Drosophila* cells, the protein known as *D. melanogaster* calcium-binding protein 1 (DmCaBP1) was discovered. This protein is located in the endoplasmic reticulum and is expressed throughout *Drosophila* development. It contains a structural domain that is similar to thioredoxin. DmCaBP1 is externalized to the cell surface in a caspase-dependent manner after apoptosis is initiated and functions as a tethering or bridge protein connecting apoptotic and phagocytic cells to promote phagocytosis. Both PRTP and DmCaBP1 are required for Draper-mediated phagocytosis, with the former in charge of activating Draper and the latter linking apoptotic and phagocytic cells (Okada et al., 2012). A recent study discovered that NimB4, a protein secreted by the Nimrod family, binds to apoptotic receptors in *D. melanogaster* in a PS-dependent manner and promotes phagocytosis of apoptotic cells by glial cells and macrophages. NimB4 is involved in phagosome maturation, and NimB4 mutant macrophages exhibit defects in phagocytosis and accumulate apoptotic cells in both embryonic and larval phagocytes, resulting in more severe larval movement and a shortened adult lifespan. This study reveals that NimB4 may act as a bridging molecule in the clearance of apoptotic cells in *D. melanogaster*, and it also demonstrates that bridging molecules have been conserved in phagocytosis as shown in Figure 1 (Petrignani et al., 2021).

### 3.3 Mammals secreted proteins in apoptotic cell clearance

Platelet-responsive protein TSP-1, the prototypical thrombin-responsive protein, is a calcium-binding glycoprotein of 450 kD with a complex functional profile. *In vivo*, TSP-1 inhibits angiogenesis by causing the death of endothelial cells involved in blood vessel formation. This is accomplished by enhancing the quantity of Fas/CD95 receptors on the surface of endothelial cells. This inhibits angiogenesis by preventing the production of FasL, which is essential for causing endothelial cell apoptosis and inhibiting angiogenesis (Volpert et al., 2002). Consequently, TSP-1 is specific to vascular remodeling. Human monocyte-derived macrophages recognize senescent neutrophils through the function of TSP and the macrophage surface molecule CD36, which acts as a molecular bridge between apoptotic neutrophils and macrophages. On the surface of macrophages, TSP creates an adhesion com-plex composed of macrophage CD36 and Vß3 integrins, which act synergistically on macrophage recognition of apoptotic neutrophil granulocytes (Silverstein and Febbraio, 2009). TSP-1 promotes the migration and phagocytosis of apoptotic cells by monocyte-derived macrophages and lymphoma cells. In pathological conditions characterized by a large number of circulating unidentified deceased cells, elevated levels of endothelium-derived TSP-1 may serve as an attractive signal for phagocytosis, thereby facilitating the recognition and clearance of apoptotic cells. The selective expression of TSP-1 as an inhibitory mediator in the myocardial infarct region restricts the extension of the inflammatory response to the non-infarct region, and the influence of TSP-1 on the myocardial infarct healing process in terms of inflammation and fibrosis.

**FIGURE 2:**
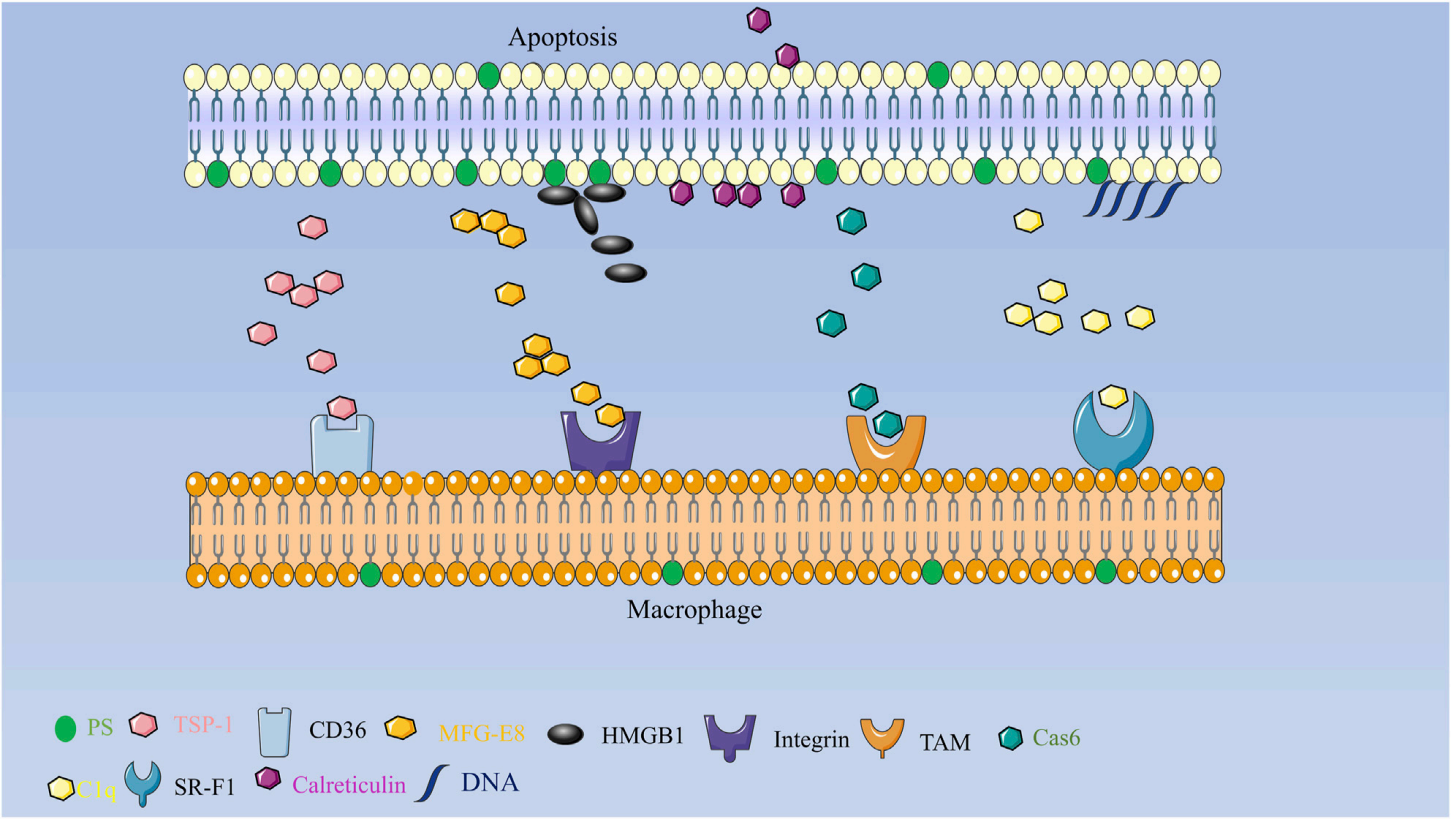
Schematic diagram of the mechanism of secreted proteins involved in apoptotic clearance in mammals TSP forms an adhesion complex containing macrophage CD36 and αVβ3 integrins that act synergistically on macrophage phagocytosis of apoptotic cells; MFG-E8 binds specifically to apoptotic cells by recognizing aminophospholipids such as PS and PE; TAM receptor tyrosine kinase together with its ligand Gas6 drives phagocyte recognition of apoptotic cells; scavenger receptor SR-F1 recognizes and phago-cytoses apoptotic cells through the complement component C1q recognizes and phagocytizes apoptotic cells, and HMGB1 can bind to PS on apoptotic neutrophils and will inhibit phagocytosis by macrophages.

It is possible that TSP-1’s inhibitory effect on inflammatory and fibrotic processes during myocardial infarction healing is due to its activation of transforming growth factor or inhibition of angiogenesis. TSP-1 leads to a phagocytic and tolerogenic phenotype in dendritic cell DCs. The N-terminal portion of TSP-1 - heparin-binding structural domain (HBD) is cleaved and secreted simultaneously in a caspase- and serine protease-dependent manner. tsp-1 or HBD binds primarily to mucous cell IDCs or phagocytosis, triggering the formation of signaling vesicles that enhance phagocytosis, mediate inhibitory signals that leading to a tolerogenic phenotype and possibly the formation of bridging molecules, apoptotic cells then bind to phagocytes via TSP-1 and its associated receptors such as CD91/calreticulin (LRP), CD36, and αVβ3, and are subsequently engulfed as shown in Figure 2 (Mikhailenko et al., 1997; Krispin et al., 2006; Tabib et al., 2009).

Milk fat globular epidermal growth factor 8 (MFG-E8) is a secreted protein that is made by thioglycolic acid-activated macrophages (Lu et al., 2021). It acts as a bridge by recognizing aminophospholipids like PS and PE that bind to apoptotic cells and carry them to phagocytes. Its RGD (arginine-glycine-aspartate) motif is then recognized by some members of the integrin family. MFG-E8-deficient mice develop splenomegaly, form many germinal centers, and develop glomerulonephritis due to autoantibody production. MFG-E8 deficiency leads to impaired clearance of apoptotic cells and accumulation in mice after myocardial infarction, leading to an increased inflammatory response and a dramatic de-crease in survival, whereas MFG-E8 administration after myocardial infarction restores cardiac function and cardiac morphology. These phenomena suggest that MFG-E8 plays a key role in the clearance of apoptotic cells and that its downregulation of expression di-minishes the phagocytic capacity of macrophages, which may contribute to the development of inflammatory and autoimmune diseases (Hanayama et al., 2004b). In addition, MFG-E8 reduces neutrophil migration and network aggregation by downregulating CXCR2 expression on the neutrophil surface while also playing a role in neutrophil phagocytosis of apoptotic cells (Aziz et al., 2015). MFG-E8 is structurally and functionally similar to the developing endothelial gene Del-1. Both are made up of a signal sequence, two epidermal growth factor structural domains, and two factor VIII-homologous structural domains (C1 and C2), which are ex-pressed in the resident and inflammatory subpopulations of macrophages, respectively (Hanayama et al., 2006). Similar to MFG-E8, Del-1 binds with high affinity to PS exposed on the surface of apoptotic cells and is recognized by phagocyte surface-associated integrin αVβ3, which acts as a bridge between apoptotic and phagocytic cells as shown in Figure 2 (Hanayama et al., 2004a). In addition to this, Del-1 regulates neutrophil production and recruitment in different ways, and in the bone marrow, Del-1 interacts with β3 integrins on hematopoietic stem cells (HSCs) to induce HSC proliferation and biased differentiation to the myeloid lineage (MYP, myeloid progenitor cells) (Mitroulis et al., 2017). In the vascular lumen, Del-1 blocks the interaction between the LFA-1 (αLβ2) integrin on neutrophils and the intercellular adhesion molecule ICAM-1 on vascular endothelial cells, thereby inhibiting neutrophil adhesion and migration. Del-1 has a dual mode of action in the inflammatory process through the different structural domains and regionalized ex-pression of its molecules, both regulating inflammatory cell recruitment and promoting the formation of a macrophage phenotype that supports catabolism (Hajishengallis and Sahingur, 2014). Both actions together can be therapeutically exploited to suppress destructive inflammation and mediate the reestablishment of tissue integrity (Kourtzelis et al., 2019).

Microglia are tissue macrophages of the central nervous system (CNS). They play an important role in CNS endostasis and are responsible for the routine, non-inflammatory removal of dead cells. This process is usually driven by the TAM (Tyro3/Axl/MERTK) receptor tyrosine kinase and its ligand Gas6 (growth arrest-specific protein 6), which is found in many human tissues and controls biological processes like cell proliferation (Post et al., 2021). In patients with hepatitis, serum levels of Gas6 and Axl are significantly elevated, and it is hypothesized that Gas6/Axl play a key role in fibrosis formation and chronic liver disease progression. In the field of cancer research, Gas6 binds to TAM receptors in cancer cells to promote their proliferation and migration and inhibit apoptosis; meanwhile, tumors can stimulate the overexpression and secretion of Gas6 from macrophages within the tumor by secreting interleukin IL-10 and M-CSF (macrophage colony-stimulating factor) into the microenvironment (D'Oronzo et al., 2019). Gas6 binds to the surface of NK cells (natural killer cells). Gas6 binds to the TAM receptor on the surface of NK cells (natural killer cells) and inhibits their anti-tumor immune effects (D'Oronzo et al., 2019). In addition, Gas6 also binds to TAM receptors on VSMCs (vascular smooth muscle cells) and promotes the proliferation and angiogenesis of VSMCs (Zhou et al., 2021). Based on the above studies, targeting Gas6/TAM pathway inhibition would provide targets for inflammation and cancer therapy. Recent studies have revealed that Gas6 may have a protective role in BLM-induced lung disease. Exogenous administration of recombinant Gas6 to mice reduces the secretion of pro-inflammatory cytokines and slows inflammation and apoptosis, while combined treatment with BLM and rGas6 enhances phagocytosis of apoptotic cells by alveolar macrophages in mice (Zhou et al., 2021). Numerous complement proteins function as crucial modulators to enable phagocyte detection and clearance of apoptotic and necrotic cells.

The complement system is an essential component of innate immunity. To exert regulatory effects and promote the phagocytosis of antigens, complement can adhere to the surface of phagocytes. C1q, C1r, and C1s all make up the initial response protein known as complement protein C1. A variety of cells, including macrophages, epithelial cells, dendritic cells, *etc.*, can release C1q, a hexameric plasma glycoprotein (Espericueta et al., 2020). An significant risk factor for systemic lupus erythematosus (SLE) in humans is inherited C1q deficiency. Through the complement component C1q, the scavenger receptor SR-F1 recognizes and phagocyte apoptotic cells. The N-terminal end of the extracellular region of SR-F1 interacts with the collagen tail of C1q, while the globular head of C1q binds to “Eat-Me” signals like PS, DNA, or the calcium reticulum protein CRT (Wicker-Planquart et al., 2020). Extracellular vesicles (EV) are released by non-inflammatory macrophages with the aid of the pattern recognition receptor (PRR) C1q, which acts as an anchoring scaffold for C1r and C1s protein hydrolases to initiate the classical complement cascade and produce pro-inflammatory division products (Yan and Bai, 2019). C1q participates in a number of pathogenic processes in neurodegenerative illnesses through activation, microglia binding, and activation. Recent research has shown that the expression of protein hydrolase 3 (PR3) on apoptotic cells inhibits C1q-mediated phagocytosis. Chronic inflammatory illnesses are made worse by the membrane expression of PR3, a pro-inflammatory factor. Without membrane PR3, C1q regulates apoptotic cells via apoptotic cell-associated molecular pattern recognition, improves phagocytosis via macro-phage receptors, and triggers an anti-inflammatory response that leads to inflammation remission. When membrane PR3 is present, PR3 found on dying cells binds PS, CRT, and C1q as shown in Figure 2. This can disrupt the multimolecular complex at the junction of apoptotic neutrophils and macrophages, delaying or impairing the phagocytosis of apoptotic cells and activating an inflammatory immune response that promotes autoimmunity (Tacnet-Delorme et al., 2018). As bridging molecules, secretory proteins can promote phagocytosis, and certain secretory proteins interact with “Eat-Me” signals and phagocytic receptors to inhibit phagocytosis in apoptotic cells. It has been demonstrated that HMGB1 binds to PS on apoptotic cells and inhibits macrophage phagocytosis. It is hypothesized that HMGB1 may inhibit the recognition of apoptotic cells by phagocytes by covering PS on the surface of apoptotic cells (Chen et al., 2004; Liu et al., 2008; Friggeri et al., 2010; Yang et al., 2022).

### 3.4 Secreted proteins that are involved in apoptotic cell clearance and its association with various human diseases

Secreted proteins engaged in apoptotic cell clearance play an important role in hu-man diseases. Clinical studies have shown that aberrant TSP1 expression is linked to obesity, fatty liver disease, and diabetes (Roberts and Isenberg, 2021). NETs play a role in the pathophysiology of HFpEF, which can be treated with HMGB1 and SGLT2 inhibitors. HMGB1 and NETs could be new therapeutic targets for the treatment of HFpEF (Zhang et al., 2022). Milk Fat Globule Epi-dermal Growth Factor 8 (MFGE8) deficiency and gene polymorphisms have previously been associated to SLE-like and SLE development (Skarlis et al., 2022). The GAS6-PROS1/TAM system has been linked to SARS-CoV-2 infection、COVID-19 (Tutusaus et al., 2020)、Systemic lupus erythematosus (Cohen and Shao, 2019) and multiple sclerosis (Li et al., 2020). C1q is involved in synapse creation and pruning, and it is a crucial actor in the development and homeostasis of neuronal networks in the CNS. C1q has a close association with microglia and astrocytes, and it may contribute to the development of CNS illnesses under their influence (Zhang et al., 2023). Although these released proteins have been associated with a number of human disorders, more research is needed to determine how the secreted proteins influence the development of these diseases.

## 4 Discussion

The prompt and efficient removal of apoptotic cells is vital for maintaining the organism’s equilibrium. Having compared secreted proteins associated with apoptotic cell clearance in *Drosophila*, nematodes, and mammals, the function of secreted proteins in apoptotic cell clearance are highly conserved in model organisms and in mammals (Zhang et al., 2022). Impaired synthesis of secretory proteins may result in the deposition and accumulation of aggregation-prone proteins or drastically alter the release and concentration of other secretory proteins during apoptotic cell clearance (Gonzalez et al., 2020). In this article, we discuss the role of secreted proteins that have been found in the clearance of apoptotic cells in mammals, *Drosophila melanogaster*, and Cryptobacterium histolytica, although the precise mode of action is yet unknown. It has been found that secreted proteins, which are predominantly produced through the classical secretory protein route, perform a crucial role in the recruitment and recognition of apoptotic cells during clearance, principally acting as bridging molecules between apoptotic cells and phagocytes. In other reports on secretory proteins, it was discovered that secretory proteins act intracellularly or extracellularly through translational modifications, that different modifications affect the function of secretory proteins, and that secretory kinases and phosphatases regulate secretory proteins during phase transitions (Young et al., 2020). Cytokines, as a class of classically secreted proteins, are soluble mediators of immune and inflammatory responses and are externalized through the classical endoplasmic reticulum (ER)-Golgi extravasation pathway (Dinarello, 2000). However, some lack signal peptides and are secreted through non-classical secretory mechanisms (Rubartelli and Sitia, 1997). The prototype of leaderless cytokines is interleukin (IL)-1β, the major proinflammatory factor produced by activated monocytes. IL-1β is partially translocated from the cytosol, where it aggregates into secretory lysosomes and is secreted in exogenous ATP-induced exocytosis of these organelles (Andrei et al., 1999). Secretory lysosomes are secretory organelles regulated by Ca2+ and display the characteristics of lysosomes and secretory granules. It has also been reported that in mice insulin secretion is regulated by changes in ion channels (Pang et al., 2020). The study of secreted proteins in-volved in apoptotic cell clearance, along with protein modifications, ion channel regulation, inflammatory immunity, and other potential mechanisms of action, will enable us to comprehend the mechanisms of action of apoptotic cell clearance.
